# 1,3-Dithian-2-one azine

**DOI:** 10.1107/S1600536810010524

**Published:** 2010-03-27

**Authors:** Yan-Bo Wang, Yan Shi, Xiao-Lan Liu, Yong-Hong Liu

**Affiliations:** a College of Life Science and Chemistry, Tianshui Normal University, Tianshui 741000, People’s Republic of China; b College of Chemistry and Chemical Engineering, Yangzhou University, Yangzhou, 225002, People’s Republic of China

## Abstract

In an asymmetric unit of the title compound, C_8_H_12_N_2_S_4_, there are two crystallographically independent half mol­ecules lying on inversion centers. One of the mol­ecules is disordered over two positions with relative occupancies of 82.0 (2) and 18.0 (2) for the major and minor components. In the crystal structure, mol­ecules are linked into a three-dimensional framework *via* inter­molecular C—H⋯N hydrogen-bonding inter­actions.

## Related literature

For the synthesis, see: Mayer & Schaefer (1964 ▶); Xu *et al.* (2005 ▶). For the use of 2-hydrazono-1,3-dithiol­ane derivatives in coordination chemistry and their biological activity, see: Beghidja *et al.* (2006 ▶); Gou *et al.* (2004 ▶). For 1,3-dithian-2-yl­idene derivatives as anti­mycotic agents and an important synthesis medium, see: Dong *et al.* (2005 ▶); Ram *et al.* (1997 ▶). For related structures, see: Liu, Liu & Liu (2008 ▶); Liu, Liu, Dai *et al.* (2008 ▶); Yang *et al.* (2007 ▶). For graph-set notation, see: Bernstein *et al.* (1995 ▶). For dithian ring conformations, see: Boeyens (1978 ▶).
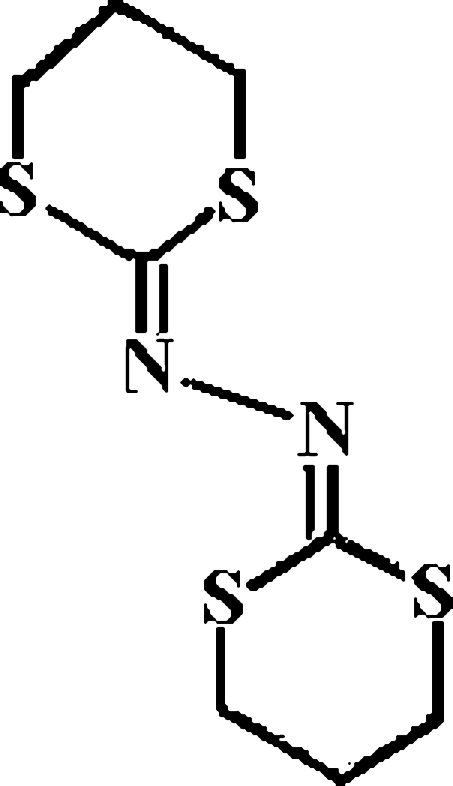


## Experimental

### 

#### Crystal data

C_8_H_12_N_2_S_4_
*M* *_r_* = 264.44
Monoclinic, 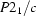

*a* = 9.3999 (11) Å
*b* = 11.9251 (14) Å
*c* = 10.7397 (13) Å
β = 91.555 (2)°
*V* = 1203.4 (2) Å^3^
*Z* = 4
Mo *K*α radiation
μ = 0.75 mm^−1^
*T* = 293 K
0.22 × 0.21 × 0.19 mm

#### Data collection

Bruker SMART 1000 CCD diffractometer
Absorption correction: multi-scan (*SADABS*; Bruker, 2002 ▶) *T*_min_ = 0.852, *T*_max_ = 0.870
10905 measured reflections
2998 independent reflections
2478 reflections with *I* > 2σ(*I*)
*R*_int_ = 0.055

#### Refinement

*R*[*F* ^2^ > 2σ(*F* ^2^)] = 0.046
*wR*(*F* ^2^) = 0.127
*S* = 1.01
2998 reflections
151 parameters
14 restraints
H-atom parameters constrained
Δρ_max_ = 0.65 e Å^−3^
Δρ_min_ = −0.41 e Å^−3^

### 

Data collection: *SMART* (Bruker, 2002 ▶); cell refinement: *SAINT* (Bruker, 2002 ▶); data reduction: *SAINT*; program(s) used to solve structure: *SHELXS97* (Sheldrick, 2008 ▶); program(s) used to refine structure: *SHELXL97* (Sheldrick, 2008 ▶); molecular graphics: *PLATON* (Spek, 2009 ▶); software used to prepare material for publication: *SHELXTL* (Sheldrick, 2008 ▶).

## Supplementary Material

Crystal structure: contains datablocks I, global. DOI: 10.1107/S1600536810010524/pv2263sup1.cif

Structure factors: contains datablocks I. DOI: 10.1107/S1600536810010524/pv2263Isup2.hkl

Additional supplementary materials:  crystallographic information; 3D view; checkCIF report

## Figures and Tables

**Table 1:** Hydrogen-bond geometry (Å, °)

*D*—H⋯*A*	*D*—H	H⋯*A*	*D*⋯*A*	*D*—H⋯*A*
C4—H4*B*⋯N2^i^	0.97	2.71	3.620 (7)	157
C4′—H4*C*⋯N2^i^	0.97	2.70	3.503 (7)	141
C6—H6*B*⋯N1^i^	0.97	2.62	3.397 (6)	137
C6—H6*B*⋯N1′^ii^	0.97	2.68	3.428 (6)	134
C2′—H2*D*⋯N2^iii^	0.97	2.64	3.529 (7)	152
C2—H2*B*⋯N2^iii^	0.97	2.78	3.523 (7)	134
C8—H8*B*⋯N1^iv^	0.97	2.73	3.654 (3)	159
C8—H8*B*⋯N1′	0.97	2.70	3.591 (3)	154

Symmetry codes: (i) 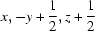; (ii) 
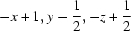
; (iii) 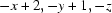; (iv) 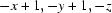.

**Fig. 1.**
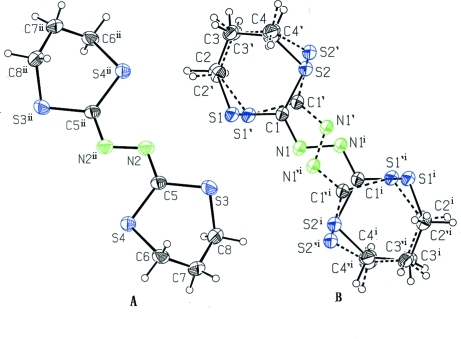
The two independent molecules of the title compound, showing 30% probability ellipsoids; minor fraction of molecule B has been plotted with dashed lines. The symmetry codes are: (i) x+1, y+1, z; (ii) x+2, y, z].

**Fig. 2.**
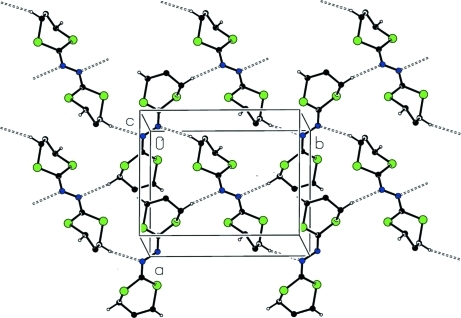
Unit cell packing of the title compound, showing the formation of a sheet in the ab plane. The H atoms not involved in hydrogen bonding have been omitted for clarity.

**Fig. 3.**
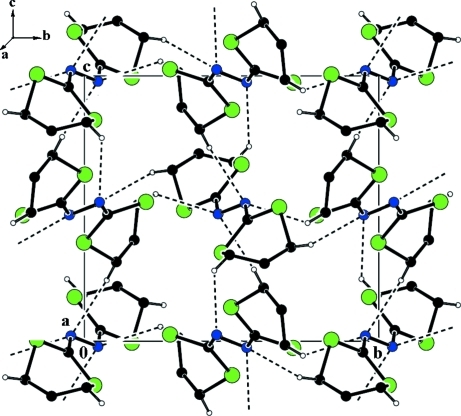
Unit cell packing of the title compound, showing three dimensional frame work as a result of C–H···N inter-molecular hydrogen-bonds (as dashed lines) viewed along the a axis. The H atoms not involved in hydrogen bonding have been omitted for clarity.

## supplementary crystallographic information

### Comment

The derivatives of 2-hydrazono-1,3-dithiolane have been abstracted for their coordination chemistry and biological activities (Beghidja *et al.*, 2006; Gou *et al.*, 2004). The derivatives of 1,3-dithian-2-ylidene and thiazolidin-2-ylidene are a novel class of antimycotic agents and important synthesis mediam (Ram *et al.*, 1997; Dong *et al.*, 2005). But very few derivatives of 2-hydrazono-1,3-dithiane have been reported. As on going research in this field in our laboratory (Liu *et al.*, 2008; Yang *et al.*, 2007), we report herein the structure of the title compound.

In the title compound, there are two crystallographically independent half molecules in the asymmetric unit, which lie on centres of symmetry; referred as molecules A and B. The atoms of the molecule B are disordered over two positions with relative occupancies of 82.0 (2) and 18.0 (2) for the major and minor components, respectively. All the dithian rings adopt twist-boat conformations (Boeyens, 1978). The atoms S1/S2/C1/C3/N1 in molecule A lie in a plane and the atoms C2 and C4 lie above and below this plane. Similarly, atoms S3/S4/C5/C7/N2 in molecule B also lie in a plane and the atoms C6 and C8 lie above and below the plane. The molecular dimensions  in the two molecules are similar with the corresponding molecular dimensions reported in similar structures from our previous work (Yang *et al.*., 2007; Liu, Liu & Liu, (2008); Liu, Liu, Dai *et al.*, (2008).

In the crystal structure the molecules are joined into a zig-zag chain by C8–H8B···N1 inter-molecular hydrogen-bond (Fig. 2, Tab. 1). At the same time C2–H2B···N2 inter-molecular hydrogen-bonds form another zig-zag chain along the molecular long axis and vertically the first chain. Both of them generate a sheet with edge-fused R_4_^4^(22) rings in graph set notation (Bernstein *et al.*, 1995) in the *ab*-plane. Besides these chains, there are two more zig-zag chains, formed by C6–H6B···N1 and C4–H4B···N2 inter-molecular hydrogen-bonds which make up the adjacent sheet into a three dimensional frame work along the *c* axis (Fig. 3).

### Experimental

The title compound was prepared according to the reference method (Mayer & Schaefer, 1964; Xu *et al.*, 2005) and crystallized from a mixture of ethanol and petroleum ether (1:8).

### Refinement

The atoms of the molecule B are disordered over two positions with relative occupancies of 82.0 (2) and 18.0 (2) for the major and minor components, respectively; their anisotropic dispalcement parameters were constrained to be equal. Restraints were applied to bond distances in the disordered molecule B in reference to the molecule A. All H atoms were placed at ideal positions and allowed to ride on the parent C atoms, with C–H = 0.97 and *U*_iso_(H) values of 1.2*U*_eq_(C).

### Crystal data

**Table:**

C_8_H_12_N_2_S_4_	*F*(000) = 552
*M**_r_* = 264.44	*D*_x_ = 1.460 Mg m^−^^3^
Monoclinic, *P*2_1_/*c*	Mo *K*α radiation, λ = 0.71073 Å
Hall symbol: -P 2ybc	Cell parameters from 5734 reflections
*a* = 9.3999 (11) Å	θ = 2.8–28.2°
*b* = 11.9251 (14) Å	µ = 0.75 mm^−^^1^
*c* = 10.7397 (13) Å	*T* = 293 K
β = 91.555 (2)°	Block, colorless
*V* = 1203.4 (2) Å^3^	0.22 × 0.21 × 0.19 mm
*Z* = 4	

### Data collection

**Table:**

Bruker SMART 1000 CCD diffractometer	2998 independent reflections
Radiation source: fine-focus sealed tube	2478 reflections with *I* > 2σ(*I*)
graphite	*R*_int_ = 0.055
φ & ω scans	θ_max_ = 28.4°, θ_min_ = 2.2°
Absorption correction: multi-scan (*SADABS*; Bruker, 2002)	*h* = −12→12
*T*_min_ = 0.852, *T*_max_ = 0.870	*k* = −15→15
10905 measured reflections	*l* = −14→14

### Refinement

**Table:**

Refinement on *F*^2^	Primary atom site location: structure-invariant direct methods
Least-squares matrix: full	Secondary atom site location: difference Fourier map
*R*[*F*^2^ > 2σ(*F*^2^)] = 0.046	Hydrogen site location: inferred from neighbouring sites
*wR*(*F*^2^) = 0.127	H-atom parameters constrained
*S* = 1.01	*w* = 1/[σ^2^(*F*_o_^2^) + (0.0561*P*)^2^ + 0.7487*P*] where *P* = (*F*_o_^2^ + 2*F*_c_^2^)/3
2998 reflections	(Δ/σ)_max_ < 0.001
151 parameters	Δρ_max_ = 0.65 e Å^−^^3^
14 restraints	Δρ_min_ = −0.41 e Å^−^^3^

### Special details

**Table:**

Geometry. All esds (except the esd in the dihedral angle between two l.s. planes) are estimated using the full covariance matrix. The cell esds are taken into account individually in the estimation of esds in distances, angles and torsion angles; correlations between esds in cell parameters are only used when they are defined by crystal symmetry. An approximate (isotropic) treatment of cell esds is used for estimating esds involving l.s. planes.
Refinement. Refinement of *F*^2^ against ALL reflections. The weighted *R*-factor wR and goodness of fit *S* are based on *F*^2^, conventional *R*-factors *R* are based on *F*, with *F* set to zero for negative *F*^2^. The threshold expression of *F*^2^ > σ(*F*^2^) is used only for calculating *R*-factors(gt) etc. and is not relevant to the choice of reflections for refinement. *R*-factors based on *F*^2^ are statistically about twice as large as those based on *F*, and *R*- factors based on ALL data will be even larger.

### Fractional atomic coordinates and isotropic or equivalent isotropic displacement parameters (Å^2^)

**Table:**

	*x*	*y*	*z*	*U*_iso_*/*U*_eq_	Occ. (<1)
S1	0.71338 (9)	0.70901 (9)	−0.02533 (9)	0.0596 (3)	0.820 (2)
S2	0.74955 (10)	0.49843 (7)	0.12799 (9)	0.0510 (2)	0.820 (2)
N1	0.5250 (2)	0.5510 (2)	−0.0239 (2)	0.0424 (5)	0.820 (2)
C1	0.6487 (3)	0.5788 (2)	0.0219 (2)	0.0373 (5)	0.820 (2)
C2	0.9037 (4)	0.6810 (11)	−0.0147 (11)	0.0581 (12)	0.820 (2)
H2A	0.9235	0.6125	−0.0598	0.070*	0.820 (2)
H2B	0.9540	0.7416	−0.0545	0.070*	0.820 (2)
C3	0.9597 (16)	0.6690 (9)	0.1184 (11)	0.0525 (15)	0.820 (2)
H3A	1.0489	0.6282	0.1185	0.063*	0.820 (2)
H3B	0.9787	0.7430	0.1525	0.063*	0.820 (2)
C4	0.8569 (18)	0.6085 (10)	0.2011 (13)	0.0554 (15)	0.820 (2)
H4A	0.7930	0.6637	0.2355	0.053 (9)*	0.820 (2)
H4B	0.9109	0.5758	0.2702	0.072 (12)*	0.820 (2)
S1'	0.6929 (5)	0.6622 (4)	−0.0158 (5)	0.0596 (3)	0.180 (2)
S2'	0.7925 (5)	0.4809 (4)	0.1612 (5)	0.0510 (2)	0.180 (2)
N1'	0.5477 (10)	0.4742 (9)	0.0420 (10)	0.0424 (5)	0.180 (2)
C1'	0.6632 (10)	0.5345 (8)	0.0577 (10)	0.0373 (5)	0.180 (2)
C2'	0.8826 (16)	0.687 (5)	−0.007 (6)	0.0581 (12)	0.180 (2)
H2C	0.9253	0.6486	−0.0756	0.070*	0.180 (2)
H2D	0.8981	0.7670	−0.0190	0.070*	0.180 (2)
C3'	0.962 (8)	0.653 (5)	0.112 (5)	0.0525 (15)	0.180 (2)
H3D	1.0285	0.5940	0.0920	0.063*	0.180 (2)
H3C	1.0171	0.7168	0.1423	0.063*	0.180 (2)
C4'	0.868 (9)	0.613 (4)	0.215 (7)	0.0554 (15)	0.180 (2)
H4D	0.7935	0.6667	0.2298	0.066*	0.180 (2)
H4C	0.9236	0.6018	0.2911	0.066*	0.180 (2)
S3	0.72455 (7)	0.16120 (6)	−0.00561 (6)	0.0599 (2)	
S4	0.79852 (6)	−0.03342 (5)	0.16643 (6)	0.04884 (18)	
N2	0.9581 (2)	0.04587 (17)	−0.02005 (18)	0.0489 (5)	
C5	0.8418 (2)	0.05424 (19)	0.0413 (2)	0.0428 (5)	
C6	0.6094 (2)	−0.0073 (2)	0.1744 (2)	0.0541 (6)	
H6A	0.5649	−0.0259	0.0945	0.065*	
H6B	0.5702	−0.0570	0.2362	0.065*	
C7	0.5708 (4)	0.1115 (3)	0.2068 (4)	0.0831 (11)	
H7A	0.5798	0.1205	0.2965	0.100*	
H7B	0.4717	0.1239	0.1831	0.100*	
C8	0.6605 (3)	0.2012 (2)	0.1454 (3)	0.0649 (7)	
H8A	0.7415	0.2185	0.1999	0.078*	
H8B	0.6041	0.2690	0.1361	0.078*	

### Atomic displacement parameters (Å^2^)

**Table:**

	*U*^11^	*U*^22^	*U*^33^	*U*^12^	*U*^13^	*U*^23^
S1	0.0474 (4)	0.0466 (6)	0.0836 (5)	−0.0132 (4)	−0.0182 (4)	0.0194 (4)
S2	0.0423 (5)	0.0480 (4)	0.0625 (5)	−0.0055 (3)	−0.0045 (3)	0.0150 (3)
N1	0.0389 (12)	0.0444 (12)	0.0440 (11)	−0.0099 (10)	0.0005 (9)	0.0025 (9)
C1	0.0360 (11)	0.0375 (14)	0.0385 (13)	−0.0038 (11)	0.0038 (9)	−0.0002 (9)
C2	0.0420 (16)	0.069 (2)	0.063 (2)	−0.015 (3)	0.002 (2)	0.0097 (18)
C3	0.0398 (14)	0.048 (4)	0.069 (2)	−0.008 (3)	−0.0076 (15)	0.002 (2)
C4	0.044 (3)	0.0705 (19)	0.051 (4)	−0.0066 (13)	−0.006 (3)	−0.0005 (16)
S1'	0.0474 (4)	0.0466 (6)	0.0836 (5)	−0.0132 (4)	−0.0182 (4)	0.0194 (4)
S2'	0.0423 (5)	0.0480 (4)	0.0625 (5)	−0.0055 (3)	−0.0045 (3)	0.0150 (3)
N1'	0.0389 (12)	0.0444 (12)	0.0440 (11)	−0.0099 (10)	0.0005 (9)	0.0025 (9)
C1'	0.0360 (11)	0.0375 (14)	0.0385 (13)	−0.0038 (11)	0.0038 (9)	−0.0002 (9)
C2'	0.0420 (16)	0.069 (2)	0.063 (2)	−0.015 (3)	0.002 (2)	0.0097 (18)
C3'	0.0398 (14)	0.048 (4)	0.069 (2)	−0.008 (3)	−0.0076 (15)	0.002 (2)
C4'	0.044 (3)	0.0705 (19)	0.051 (4)	−0.0066 (13)	−0.006 (3)	−0.0005 (16)
S3	0.0493 (4)	0.0688 (5)	0.0621 (4)	0.0031 (3)	0.0130 (3)	0.0238 (3)
S4	0.0423 (3)	0.0489 (3)	0.0557 (3)	−0.0007 (2)	0.0075 (2)	0.0089 (2)
N2	0.0451 (10)	0.0511 (11)	0.0511 (10)	−0.0027 (8)	0.0109 (8)	0.0014 (8)
C5	0.0382 (10)	0.0455 (11)	0.0449 (11)	−0.0057 (9)	0.0069 (8)	−0.0018 (9)
C6	0.0391 (11)	0.0677 (16)	0.0560 (13)	−0.0061 (11)	0.0097 (10)	0.0143 (11)
C7	0.0716 (19)	0.080 (2)	0.100 (2)	0.0246 (17)	0.0506 (18)	0.0327 (18)
C8	0.0587 (15)	0.0575 (15)	0.0798 (18)	0.0140 (13)	0.0237 (13)	0.0095 (13)

### Geometric parameters (Å, °)

**Table:**

S1—C1	1.748 (3)	C2'—H2C	0.9700
S1—C2	1.821 (5)	C2'—H2D	0.9700
S2—C1	1.749 (3)	C3'—C4'	1.512 (10)
S2—C4	1.821 (4)	C3'—H3D	0.9700
N1—C1	1.293 (3)	C3'—H3C	0.9700
N1—N1^i^	1.406 (4)	C4'—H4D	0.9700
C2—C3	1.516 (5)	C4'—H4C	0.9700
C2—H2A	0.9700	S3—C5	1.751 (2)
C2—H2B	0.9700	S3—C8	1.810 (3)
C3—C4	1.514 (5)	S4—C5	1.759 (2)
C3—H3A	0.9700	S4—C6	1.809 (2)
C3—H3B	0.9700	N2—C5	1.296 (3)
C4—H4A	0.9700	N2—N2^ii^	1.409 (4)
C4—H4B	0.9700	C6—C7	1.505 (4)
S1'—C1'	1.741 (8)	C6—H6A	0.9700
S1'—C2'	1.809 (10)	C6—H6B	0.9700
S2'—C1'	1.746 (8)	C7—C8	1.523 (4)
S2'—C4'	1.811 (10)	C7—H7A	0.9700
N1'—C1'	1.309 (9)	C7—H7B	0.9700
N1'—N1'^i^	1.40 (2)	C8—H8A	0.9700
C2'—C3'	1.513 (10)	C8—H8B	0.9700
			
C1—S1—C2	99.6 (4)	C4'—C3'—C2'	115 (6)
C1—S2—C4	99.8 (6)	C4'—C3'—H3D	108.6
C1—N1—N1^i^	112.9 (3)	C2'—C3'—H3D	108.6
N1—C1—S1	115.7 (2)	C4'—C3'—H3C	108.6
N1—C1—S2	125.0 (2)	C2'—C3'—H3C	108.6
S1—C1—S2	119.30 (14)	H3D—C3'—H3C	107.6
C3—C2—S1	113.1 (9)	C3'—C4'—S2'	106 (4)
C3—C2—H2A	109.0	C3'—C4'—H4D	110.5
S1—C2—H2A	109.0	S2'—C4'—H4D	110.5
C3—C2—H2B	109.0	C3'—C4'—H4C	110.5
S1—C2—H2B	109.0	S2'—C4'—H4C	110.5
H2A—C2—H2B	107.8	H4D—C4'—H4C	108.7
C4—C3—C2	112.8 (12)	C5—S3—C8	99.00 (12)
C4—C3—H3A	109.0	C5—S4—C6	100.47 (11)
C2—C3—H3A	109.0	C5—N2—N2^ii^	112.1 (2)
C4—C3—H3B	109.0	N2—C5—S3	116.29 (17)
C2—C3—H3B	109.0	N2—C5—S4	123.96 (19)
H3A—C3—H3B	107.8	S3—C5—S4	119.75 (12)
C3—C4—S2	116.5 (8)	C7—C6—S4	114.6 (2)
C3—C4—H4A	108.2	C7—C6—H6A	108.6
S2—C4—H4A	108.2	S4—C6—H6A	108.6
C3—C4—H4B	108.2	C7—C6—H6B	108.6
S2—C4—H4B	108.2	S4—C6—H6B	108.6
H4A—C4—H4B	107.3	H6A—C6—H6B	107.6
C1'—S1'—C2'	107 (2)	C6—C7—C8	114.9 (2)
C1'—S2'—C4'	98 (3)	C6—C7—H7A	108.5
C1'—N1'—N1'^i^	111.0 (11)	C8—C7—H7A	108.5
N1'—C1'—S1'	124.3 (7)	C6—C7—H7B	108.5
N1'—C1'—S2'	116.2 (7)	C8—C7—H7B	108.5
S1'—C1'—S2'	119.4 (5)	H7A—C7—H7B	107.5
C3'—C2'—S1'	118 (4)	C7—C8—S3	113.8 (2)
C3'—C2'—H2C	107.9	C7—C8—H8A	108.8
S1'—C2'—H2C	107.9	S3—C8—H8A	108.8
C3'—C2'—H2D	107.9	C7—C8—H8B	108.8
S1'—C2'—H2D	107.9	S3—C8—H8B	108.8
H2C—C2'—H2D	107.2	H8A—C8—H8B	107.7

Symmetry codes: (i) −*x*+1, −*y*+1, −*z*; (ii) −*x*+2, −*y*, −*z*.

### Hydrogen-bond geometry (Å, °)

**Table:**

*D*—H···*A*	*D*—H	H···*A*	*D*···*A*	*D*—H···*A*
C4—H4B···N2^iii^	0.97	2.71	3.620 (7)	157
C4'—H4C···N2^iii^	0.97	2.70	3.503 (7)	141
C6—H6B···N1^iii^	0.97	2.62	3.397 (6)	137
C6—H6B···N1'^iv^	0.97	2.68	3.428 (6)	134
C2'—H2D···N2^v^	0.97	2.64	3.529 (7)	152
C2—H2B···N2^v^	0.97	2.78	3.523 (7)	134
C8—H8B···N1^i^	0.97	2.73	3.654 (3)	159
C8—H8B···N1'	0.97	2.70	3.591 (3)	154

Symmetry codes: (iii) *x*, −*y*+1/2, *z*+1/2; (iv) −*x*+1, *y*−1/2, −*z*+1/2; (v) −*x*+2, −*y*+1, −*z*; (i) −*x*+1, −*y*+1, −*z*.
